# Beyond the Limits of Conventional Coagulation Tests: A Comprehensive Overview of ACLF-Related Coagulopathies

**DOI:** 10.3390/jcm14103539

**Published:** 2025-05-18

**Authors:** Dominika Kurpiewska, Artur Kośnik, Krzysztof Bieliński, Joanna Raszeja-Wyszomirska

**Affiliations:** Department of Hepatology, Transplantology and Internal Medicine, Medical University of Warsaw, Banacha 1A Str., 02-097 Warsaw, Poland; artur.kosnik@uckwum.pl (A.K.); joanna.wyszomirska@wum.edu.pl (J.R.-W.)

**Keywords:** acute-on-chronic liver failure, liver cirrhosis, coagulation disorders, von Willebrand factor

## Abstract

Acute-on-chronic liver failure (ACLF) is a complex and severe condition marked by multiple organ failure and high short-term mortality. Coagulopathy, a key component of ACLF, is characterized by rebalanced hemostasis with both hypo- and hypercoagulable features, increasing the risk of bleeding and thrombosis. Conventional coagulation tests, including prothrombin time (PT) and platelet count, fail to fully capture the complexity of coagulation dysfunction in ACLF. Advanced diagnostic tools, like viscoelastic tests (VETs), offer a more comprehensive assessment, yet they remain limited in evaluating endothelial dysfunction and fail to account for reduced levels of anticoagulant factors. Emerging therapeutic strategies targeting coagulopathies in ACLF hold promise, but their clinical efficacy remains unclear. A more nuanced approach to diagnosing and managing coagulopathy in ACLF is needed, incorporating advanced hemostatic profiling to better inform prognosis and guide treatment decisions.

## 1. Introduction

Acute-on-chronic liver failure (ACLF) is a severe form of acutely decompensated cirrhosis characterized by the failure of at least one major organ system and high short-term mortality [1]. It is linked to an excessive systemic inflammatory response triggered by precipitants, most commonly a proven bacterial infection, a severe alcohol-related hepatitis or a gastrointestinal hemorrhage with shock. No precipitant can be identified in 35% of ACLF cases [2]. The diagnosis of ACLF is based on the European Association for the Study of the Liver–Chronic Liver Failure Consortium (EASL-CLIF-C) organ failure criteria, which take into consideration six major organ systems: liver, kidney, brain, coagulation, circulation, and respiration [3]. 28-day mortality rate increases with the number of failing organ systems, spanning from 22% in ACLF grade 1 to 77% in ACLF grade 3 [1].

As one of the six organ failures defining ACLF, coagulopathy is an important and complex factor influencing the patients’ prognosis. Simultaneously reduced production of pro- and anti-coagulation factors leads to a precarious hemostatic balance [4,5], associated with both hypo- and hypercoagulable features, and thus complications of both hemorrhagic and thrombotic nature [6]. Despite that, coagulopathy is still predominantly assessed with conventional parameters such as prothrombin time (PT), international normalized ratio (INR) and platelet count, which paint an inaccurate and one-dimensional picture of ACLF as a state of hypocoagulability [7].

In this article, we summarize the current data regarding coagulation dysfunction in ACLF. Our goal is to emphasize the need to look beyond conventional coagulation tests to appreciate all aspects of this disorder. We propose that more complex hemostatic profiling in this group of patients could lead to a more accurate assessment of prognosis and the development of optimal therapeutic strategies.

## 2. Coagulopathy in ACLF: Beyond the CLIF-C Criteria Perspective

As per CLIF-C criteria, the presence of coagulopathy in ACLF is ascertained based on the international normalized ratio (INR) derived from prothrombin time [3]. While an easily obtained and objective parameter, INR does not fully reflect the extent of coagulation dysfunction in cirrhotic patients, as it does not take into account several crucial factors [8]. PT is the time required for plasma to clot following the addition of tissue factor, reflecting the function of the extrinsic and the common pathway of coagulation. As the test is carried out using blood plasma, it does not consider the number or the function of platelets, thus also excluding the role played by elevated von Willebrand factor (vWF) levels and the impact of endothelial dysfunction. It also fails to account for reduced levels of anticoagulant factors, such as protein C, protein S and antithrombin, only considering the decrease in procoagulant factors [8,9].

Other conventional tests of hemostasis also have limited utility in the context of ACLF. The activated partial thromboplastin time (APTT) is often prolonged due to high levels of coagulation factor VIII [10,11]. However, like the PT, APTT does not account for platelet function or the presence of anticoagulant factors, limiting its clinical relevance in assessing bleeding risk in liver dysfunction [8]. Thrombocytopenia is a common finding in liver disease, primarily due to splenic sequestration resulting from portal hypertension and decreased thrombopoietin production [12]. Nevertheless, elevated levels of vWF compensate for low platelet counts [4]. Although lower platelet counts reflect the severity of portal hypertension, their direct association with bleeding risk in ACLF remains under investigation [13]. Low fibrinogen levels and unstable fibrin clot formation due to impaired synthetic capacities of the liver are typical findings in ACLF patients [8,14]. Reduced fibrinogen levels have been linked to increased bleeding risk after paracentesis and to lower survival rates in patients with gastrointestinal bleeding. However, these associations weaken after adjusting for liver disease severity [15,16]. Moreover, prophylactic administration of cryoprecipitate to raise plasma fibrinogen levels did not lead to a reduction in bleeding or mortality among critically ill patients with cirrhosis [17]. These findings suggest that fibrinogen levels reflect disease severity, rather than playing a direct role in the underlying mechanisms of bleeding in this group of patients [11].

**Figure 1:**
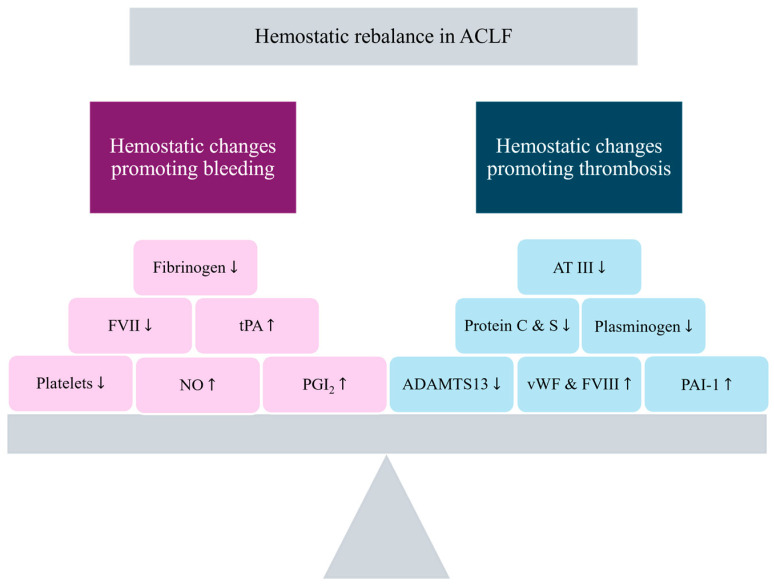
Hemostatic rebalance in ACLF. FVII—factor VII; tPA—tissue plasminogen; NO—nitric oxide; PGI2—prostacyclin; AT III—antithrombin III; vWF—von Willebrand factor; FVIII—factor VIII; PAI-1—plasminogen activator inhibitor-1. Up arrows indicate increased levels, while down arrows indicate decreased levels.

Recent evidence indicates that ACLF is a complex state of rebalanced hemostasis, characterized by various hypo- and hypercoagulable features and an increased risk of both bleeding and thrombosis (Figure 1). Therefore, it is crucial to consider both aspects while analyzing the broad spectrum of contributing factors and their interplay in a comprehensive manner [4,5,6].

## 3. A Common Misconception: Is ACLF Truly a Hypocoagulable State?

### 3.1. Whole Blood Viscoelastic Tests

Advanced laboratory assays have been designed for more adequate assessment of hemorrhage and thrombosis. Viscoelastic tests (VETs), including thromboelastography (TEG) and rotational thromboelastography (ROTEM), analyze the real-time clotting process in a whole blood sample. The basic principle of both TEG and ROTEM is similar: a blood sample, a pin and necessary reagents are inserted into the cup. Blood is then slowly rotated to replicate slow venous flow, thereby triggering coagulation. Various coagulation activators are used in both devices to provide a detailed assessment of clot formation, strength, and fibrinolysis efficiency. Both methods produce a range of test results reflecting various aspects of the hemostatic process. These parameters include clot formation time (CFT), clotting time (CT), reaction time (R), kinetics time (K), maximum amplitude (MA), maximal clot firmness (MCF), clot formation time (CFT), the alpha angle (α), and maximum lysis (ML) [18].

Due to their global character with integrated assessment of plasma, blood cells and platelets, VETs are considered superior to conventional coagulation tests as a tool for evaluation of coagulopathies in chronic liver diseases. Alterations in VETs have been shown to better correlate with bleeding risk in this group of patients [19,20], and ROTEM provided a more comprehensive assessment of hemostasis in ACLF, with MA and MCF parameters being valuable predictive factors for bleeding [21]. Nevertheless, their utility in prediction of bleeding events in ACLF is still the subject of debate.

Several studies showed that VETs identify patients with ACLF as having hypocoagulable profile and hemorrhagic tendencies. Hemostatic profiles of patients with HBV-related ACLF were compared to those of individuals with compensated chronic hepatitis B and healthy controls using TEG. Patients with ACLF demonstrated hypocoagulable states reflected by prolonged R and K times, a reduced α angle, and lower MA. Furthermore, lower MA in ACLF was associated with a higher 90-day mortality [22]. Similar TEG results were obtained in another observational cohort study. The alterations were positively correlated with both bleeding and mortality [23]. The coagulation status in ACLF was also assessed with Sonoclot VET. A significant subset of patients was characterized by a hypocoagulable state which was associated with a higher risk of bleeding. Based on these results, the authors designed a VET-based bleeding score in ACLF [24].

Despite this evidence, it has been pointed out that VETs underestimate hemostatic potential in ACLF due to several limitations, most notably their inability to assess vessel injury and endothelial components. Both ROTEM and TEG have limited sensitivity to elevated vWF levels and decreased protein C levels, both of which compensate for the lower platelet count and decreased levels of procoagulant factors in decompensated cirrhosis [25,26,27]. An observational study on 51 patients with ACLF concluded that although ROTEM indicated a hypocoagulable state, the alterations did not correlate with bleeding rates. In contrast, 90% of patients with a clearly hypocoagulable ROTEM profile did not experience bleeding, suggesting that ROTEM is not a reliable tool for identifying ACLF patients at higher risk of bleeding [28]. The results align with the findings from a multicenter, prospective cohort study of 200 patients with acute liver injury and acute liver failure. Despite significant ROTEM abnormalities indicating a severe hypocoagulable state, no association was found between ROTEM results at admission and the risk of bleeding complications during hospitalization. However, ROTEM abnormalities were proportional to disease severity [29]. Therefore, the link between more severe ROTEM abnormalities and bleeding may stem not from a hypocoagulable state, but rather from greater liver dysfunction and increased portal hypertension in patients who experienced bleeding [28].

### 3.2. Plasma-Based Global Fibrinolysis Assay

Fibrynolytic potential can be assessed using a plasma-based fibrinolysis assay in which clot formation is triggered by tissue factors, clot breakdown is initiated by tissue-type plasminogen activator, and the entire process is monitored via turbidity measurements. The assay results have been shown to correlate with the risk of both venous and arterial thrombosis in the general population [30]. Blasi et al. investigated the fibrinolytic status of patients with ACLF and its association with clinical outcomes. They observed considerable variability, with profiles ranging from hypofibrinolysis to hyperfibrinolysis. One possible explanation for this heterogeneity is the presence of sepsis, a common complication in ACLF, which is known to induce hypofibrinolysis. Moreover, ACLF is a dynamic condition, and fibrinolytic potential can shift significantly over time. For instance, a patient may transition from a hyperfibrinolytic to a hypofibrinolytic state if sepsis develops during the course of illness. Clinically, fibrinolytic potential did not differ between ACLF patients who experienced bleeding and those who did not, nor between cases of portal hypertensive bleeding and other bleeding etiologies [31]. More research is needed to understand how fibrinolytic potential in ACLF patients changes over time and how this could affect its use in patient care and outcome prediction.

### 3.3. Thrombomodulin-Modified Thrombin Generation Tests

In healthy individuals, thrombomodulin regulates thrombin generation by activating protein C, thus acting as an anticoagulant factor. However, in patients with chronic liver disease, this regulatory mechanism is impaired due to partial resistance of plasma to the effects of thrombomodulin [32]. The thrombomodulin-modified thrombin generation tests (TM-TGTs) provide a dynamic assessment of total thrombin generation during the initiation of in vitro coagulation. By incorporating thrombomodulin, TM-TGTs capture the balance between procoagulant and anticoagulant factors, reflecting the complex hemostatic alterations seen in chronic liver disease. These tests are considered one of the most reliable methods for assessing plasma coagulation potential, as they provide a comprehensive overview of the coagulation system’s functional capacity. The limitation of traditional TM-TGTs is the non-physiological, non-cellular environment in which they are performed, which does not fully replicate in vivo conditions [5,11]. To address this issue, whole blood thrombin generation tests (WB-TGTs) have been developed as a more comprehensive method for evaluating coagulation. Unlike TM-TGTs, WB-TGTs include all circulating blood cells, offering a more physiologically accurate assessment of hemostasis in patients with cirrhosis [33]. In decompensated cirrhosis, WB-TGTs tend to indicate a hypocoagulable state, contrasting with the hypercoagulable profile observed with TM-TGTs [34]. Moreover, WB-TGTs have been shown to predict major bleeding following invasive procedures, a correlation not observed with TM-TGTs. Preserved thrombin generation as measured by WB-TGTs was also associated with a reduced risk of developing ACLF. However, since patients with ACLF were excluded from the study, WB-TGT alterations in this population were not assessed. Neither of the TGTs could predict thrombotic events [35]. Prospective trials utilizing WB-TGTs could provide more detailed insights into the progression of thrombo-hemorrhagic risk in patients with ACLF and help characterize the features and prognostic significance of WB-TGT in this specific patient population.

To date, no laboratory assay has demonstrated reliability in predicting hemorrhagic or thrombotic complications in patients with ACLF. Nonetheless, the emergence of novel assays offers potential for advancing risk assessment in this area. Combining existing tools, such as TM-TGTs with platelet count and function tests, and fibrinolysis assays, may enhance the prediction of clinical events in this complex patient population [32]. Therefore, further research is needed to explore and validate the utility of such assay combinations for predicting thrombotic and hemorrhagic complications in ACLF.

## 4. Von Willebrand Factor: A Marker of Endothelial Dysfunction

vWF is a large glycoprotein involved in the process of hemostasis and a marker of endothelial injury. It is one of the main procoagulant factors, mediating platelet adhesion to sites of vascular injury and serving as a carrier protein for Factor VIII, protecting it from degradation. ADAMTS13 is a metalloproteinase responsible for vWF deactivation, thus acting as an anticoagulant factor. The interplay between these proteins plays an important role in hemostatic balance [36].

A study analyzed hemostatic parameters in patients with liver disease of various severity, comparing stable cirrhosis, acutely decompensated (AD) cirrhosis and ACLF. While all cohorts were thrombocytopenic, there were no significant differences in platelet counts. vWF levels increased, while ADAMTS13 levels decreased significantly across the cohorts, with a particularly marked difference between the AD and the ACLF groups [6]. Similar changes in vWF and ADAMTS13 levels in patients with ACLF were observed in other studies [10]. The role of vWF as a potential prognostic marker has also been assessed. It has been shown that both vWF levels and activity are independently associated with 30-day mortality in ACLF patients. They also correlate with organ failure and liver disease severity (assessed with MELD score, CLIF-C-ACLF score and Child–Pugh score), whereby this correlation is stronger in the case of vWF activity. vWF levels are most notably elevated in case of failure of organs (as per the CLIF-C-ACLF score) with particularly large amounts of endothelium—that is, the lungs, the liver and the circulatory vascular bed [37,38]. The optimal cut-off for vWF at 842% was proposed, with patients above this threshold being at a seven-times-greater risk of 30-day mortality [38]. Interestingly, changes in vWF levels in patients with decompensated cirrhosis treated with non-selective beta-blockers (NSBB) appeared to distinguish between those who benefit from NSBB therapy and have a favorable prognosis versus those with poor outcomes. Lower vWF levels were associated with a reduced risk of further decompensation, ACLF and liver-related mortality. This is most likely the result of NSBB’s positive impact on endothelial dysfunction [39].

Disbalance between vWF and ADAMTS13 has also emerged as a potential prognostic factor. An elevated ratio of vWF levels to ADAMTS13 activity has been shown to predict the development of ACLF in cirrhotic patients, and its increase correlated with the progression of ACLF grades and mortality [40,41].

## 5. Predicting ACLF Development: Potential Role of Endothelial Injury Markers

The EASL-CLIF-C criteria define ACLF based on organ failure-an end-stage manifestation of the disease-rather than on systemic inflammation, portal hypertension, and the associated endothelial injury that constitute the underlying pathophysiological mechanisms. While this organ-failure-based approach enables accurate diagnosis and mortality prediction in an already developed ACLF, it does not allow for early identification of patients at risk of developing the syndrome [3,42].

The PREDICT study, a prospective observational study, identified three distinct clinical courses among patients with acute decompensation of cirrhosis without initial ACLF. These included: (1) pre-ACLF, comprising patients who subsequently developed ACLF and exhibited the highest 1-year mortality rate (67.4%); (2) unstable decompensated cirrhosis, characterized by one or more hospital re-admissions without progression to ACLF, with a 1-year mortality of 35.6%; and (3) stable decompensated cirrhosis, defined by the absence of re-admissions or ACLF development, and associated with the lowest 1-year mortality (9.5%). The three groups displayed significant differences in the severity of systemic inflammation, with the pre-ACLF group displaying the highest grade. However, the authors were unable to develop a predictive score for ACLF development, highlighting this as a major challenge for future research [43].

Since then, several scores aimed at predicting ACLF development have been proposed by other research groups, with some models focusing on the underlying pathophysiological mechanisms. Trebicka et al. identified gene expression patterns associated with systemic inflammation and used these findings to develop the Chronic Liver Failure–Systemic Inflammation Gene (CLIF-SIG) score, a tool designed to quantify the inflammatory state in patients with AD. Notably, 80% of patients who progressed to ACLF had a CLIF-SIG score above 0.386, suggesting that this threshold may serve as a potential prognostic cut-off in future clinical evaluations. This score proved to be more sensitive than traditional biomarkers of systemic inflammation such as cytokines, white cell count, neutrophil-to-lymphocyte ratio and C-reactive protein (CRP) [44]. Zanetto et al. developed two predictive models incorporating variables independently associated with ACLF development. The original Padua model included CRP, CLIF-C AD score, and Child–Pugh stage, while the updated Padua Model 2.0 substituted CRP with presepsin (PSP), a soluble fragment of CD14 released during inflammatory responses. Both models effectively identified patients at increased risk of progression to ACLF, demonstrating sensitivity of 74.5% and 82.9%, and specificity of 83.3% and 76.7%, respectively. Additionally, in patients classified as low risk based on the CLIF-C AD score alone, PSP could discriminate between two groups at significantly different risk of ACLF development, and in patients who did not develop ACLF-between those who progressed toward unstable versus stable decompensated cirrhosis. Interestingly, the development of the Padua Model included an assessment of baseline coagulopathy, which was not found to be associated with progression to ACLF. However, the hemostatic profiling focused primarily on coagulation parameters and did not include markers of endothelial dysfunction. The analysis encompassed coagulation factors, a thrombomodulin-modified thrombin generation assay to evaluate endogenous thrombin potential (ETP) as a marker of plasmatic hypercoagulability, fibrinolytic factors, and the plasmin–antiplasmin complex as an indicator of fibrinolytic activation [45,46,47].

The ability to predict which patients will develop ACLF is crucial, as it would allow for earlier implementation of interventions that could improve survival. This includes timely treatment or the prevention of infections and other known precipitants, as well as referral of patients to clinical trials investigating potential disease-modifying therapies. Early identification could also help define inclusion criteria for future clinical trials [47,48]. These patients could also benefit from earlier liver transplant evaluation. It has been shown that 100% of patients listed prior to ACLF development were transplanted, compared to only 71% of those listed during an ACLF episode [49].

To our knowledge, none of the prognostic scores designed to predict ACLF development incorporate markers of endothelial injury. However, the findings discussed in this review underscore the significant role of endothelial dysfunction in the pathogenesis and progression to ACLF. In particular, the dynamic changes in vWF and ADAMTS13 levels across advancing stages of cirrhosis could serve as early indicators of ACLF risk, offering a potential tool for identifying patients who may benefit from closer monitoring [6,10,40,41].

## 6. Targeting Coagulopathies in ACLF: Emerging Therapeutic Strategies

Currently, liver transplantation is the only definitive treatment for end-stage liver disease [50]. There is an urgent need for optimal treatment strategies targeting coagulopathies in ACLF, both for patients on transplant waiting lists and those disqualified from the procedure [9].

Routine administration of blood products, such as fresh frozen plasma (FFP) or platelet concentrate (PC), is not recommended as bleeding prophylaxis. This stems from the fact that many bleeding incidents in cirrhosis result not from hemostatic dysfunction, but rather from portal hypertension or mechanical vessel injury acquired during invasive procedures. Lowering INR with FFP transfusions does not improve the hemostatic profile, as it supplies both pro- and anticoagulant proteins in equal amounts, but it can lead to exacerbation of portal hypertension due to volume expansion. Targeted transfusion therapy guided by viscoelastic tests (while keeping in mind their limitations) could benefit patients with spontaneous mucosal bleeding and significant coagulation abnormalities, such as reduced coagulation factor levels, platelet dysfunction, or thrombocytopenia [9]. As it remains unclear whether abnormal TEG test results predict procedural bleeding risk, the primary utility of TEG and other viscoelastic assays may lie in guiding transfusion strategies in patients with active bleeding, particularly when hemostatic failure, rather than portal hypertension, is the likely cause [27].

Although cirrhosis has been traditionally viewed as a bleeding disorder, the need for thromboprophylaxis in cirrhotic patients is becoming increasingly recognized, particularly as a prevention of portal vein thrombosis (PVT), which significantly worsens prognosis [9]. Enoxaparin has been observed to not only prevent PVT but also decrease hepatic decompensation and improve survival [51]. Furthermore, prophylactic anticoagulation of patients with cirrhosis was not associated with a significant risk of bleeding [52,53].

Another promising strategy is endothelial-targeted therapy, focusing on the vWF–ADAMTS13 axis. ADAMTS13 supplementation has been proposed as a means to restore balance within the axis and potentially improve prognosis [40]. Therapeutic plasma exchange could also achieve this by removing harmful ultra-large vWF multimers while replenishing ADAMTS13 [54]. Additionally, N-acetylcysteine has demonstrated the ability to degrade these multimers in in vitro and animal studies, though its efficacy has yet to be confirmed in clinical trials [55,56].

**Figure 2:**
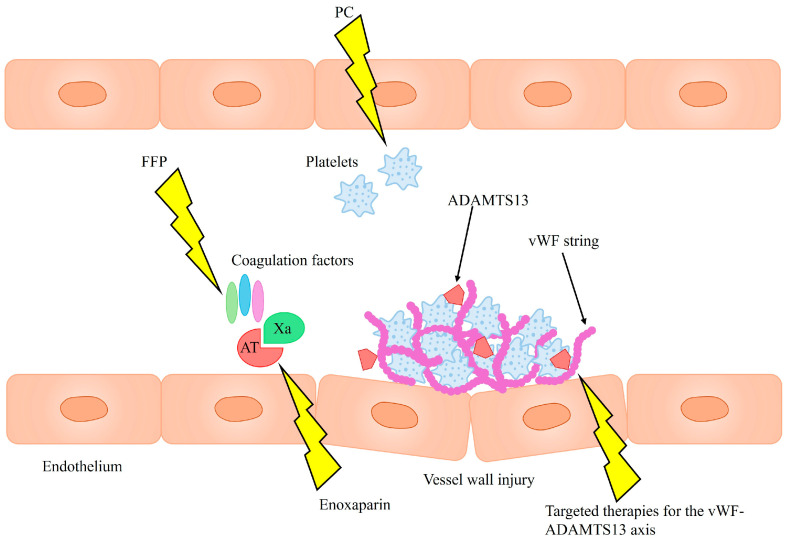
Therapeutic targets for coagulopathies in ACLF. PC—platelet concentrate; FFP—fresh frozen plasma; vWF—von Willebrand factor; AT—antithrombin; Xa—factor Xa.

The proposed therapeutic strategies are summarized in Figure 2. To date, evidence supporting the efficacy of such therapies in the context of ACLF remains limited, highlighting the need for further studies to assess their potential clinical benefits.

Future clinical trials evaluating endothelial-targeted therapies in ACLF should consider stratifying patients based on baseline levels of endothelial dysfunction markers, such as vWF and ADAMTS13. Recommended endpoints should include short-term and transplant-free survival, as well as changes in ACLF stage, with close monitoring of treatment-related adverse events, particularly bleeding and thrombotic complications [57]. Serial assessment of endothelial and inflammatory markers could help evaluate treatment response, and integrating findings with existing prognostic models could further refine patient selection and outcome prediction. Additionally, investigating the impact of early endothelial-targeted intervention in patients identified as high risk for ACLF development could determine whether timely treatment improves survival and alters the natural course of the disease [47].

## 7. Conclusions

ACLF represents a complex state of hemostatic rebalancing, rather than the traditionally assumed hypocoagulable state. Conventional coagulation tests, including PT, INR and platelet count, are insufficient in capturing the full extent of coagulation dysfunction in these patients. While viscoelastic tests, such as TEG and ROTEM, offer more comprehensive insights into the coagulation process, they have several limitations. They are not sensitive to endothelial dysfunction and certain key coagulation factors, such as vWF and ADAMTS13. They also fail to account for reduced levels of anticoagulant factors, such as protein C, protein S and antithrombin, and only consider the decrease in procoagulant factors. This often leads to an underestimation of the hemostatic potential in ACLF, which can lead to misguided treatment decisions targeting coagulopathies. A more comprehensive assessment is essential to accurately guide therapeutic strategies and improve patient outcomes. Other advanced laboratory assays such as plasma-based global fibrinolysis assay or thrombomodulin-modified WB-TGT are being tested in patients with ACLF; however, to date, none of them demonstrated reliability in predicting hemorrhagic or thrombotic complications in this clinical setting. The integration of available assays may enhance the prediction of clinical outcomes and should be the focus of future studies.

Endothelial dysfunction is closely related to organ failure and mortality in patients with ACLF. Elevated vWF levels and decreased ADAMTS13 activity are significantly associated with poor prognosis in ACLF patients, including higher 30-day mortality rates, organ failure, and disease severity. Furthermore, the interplay between vWF and ADAMTS13 provides new insights into the progression of ACLF, with an elevated vWF-to-ADAMTS13 ratio predicting ACLF development and worsening outcomes.

Early prediction of ACLF remains a major clinical challenge, as current diagnostic criteria are based on organ failure, a late-stage manifestation of the syndrome. None of the prognostic models developed to date incorporate markers of endothelial dysfunction. Given the pivotal role of endothelial injury in ACLF pathogenesis, biomarkers such as vWF and ADAMTS13 may enhance risk stratification and enable earlier interventions, including infection prevention, trial enrollment, and timely liver transplant evaluation.

Emerging evidence suggests that ACLF is characterized by both hypo- and hyper-coagulable features, which increase the risk of both bleeding and thrombosis. The importance of thromboprophylaxis is increasingly recognized, emphasizing the need for careful selection of patients who would benefit from anticoagulation therapy, while routine bleeding prophylaxis is discouraged. The vWF-ADAMTS13 axis is identified as a potential therapeutic target, with strategies aiming to restore balance in this system showing promise in improving patient outcomes. However, the clinical efficacy of these therapies remains inconclusive, and further studies are necessary to assess their potential in treating ACLF-related coagulopathies.

Overall, the current understanding of coagulopathy in ACLF requires a more complex and nuanced approach. It is essential to move beyond traditional coagulation tests and incorporate advanced diagnostic methods that consider the complexity of this disorder. Furthermore, optimal therapeutic strategies should be developed, with personalized treatment approaches guided by a thorough understanding of the hemostatic rebalance and the interplay of various contributing factors. This would enable more accurate prognosis assessment and inform the development of targeted treatments for patients with ACLF.

## Data Availability

No new data were created or analyzed in this study. Data sharing is not applicable to this article.

## Abbreviations

The following abbreviations are used in this manuscript:

ACLF	Acute-on-chronic liver failure
AD	Acutely decompensated cirrhosis
EASL-CLIF-C	European Association for the Study of the Liver–Chronic Liver Failure Consortium
PT	Prothrombin time
INR	International normalized ratio
APTT	Activated partial thromboplastin time
vWF	von Willebrand factor
VET	Viscoelastic test
TEG	Thromboelastography
ROTEM	Rotational thromboelastography
TM-TGT	Thrombomodulin-modified thrombin generation test
WB-TGT	Whole-blood thrombin generation test
NSBB	Non-selective beta-blockers

## References

[1] Moreau R., Jalan R., Gines P., Pavesi M., Angeli P., Cordoba J., Durand F., Gustot T., Saliba F., Domenicali M. (2013). Acute-on-chronic liver failure is a distinct syndrome that develops in patients with acute decompensation of cirrhosis. Gastroenterology.

[2] Trebicka J., Fernandez J., Papp M., Caraceni P., Laleman W., Gambino C., Giovo I., Uschner F.E., Jansen C., Jimenez C. (2021). PREDICT identifies precipitating events associated with the clinical course of acutely decompensated cirrhosis. J. Hepatol..

[3] Moreau R., Tonon M., Krag A., Angeli P., Berenguer M., Berzigotti A., Fernandez J., Francoz C., Gustot T., Jalan R. (2023). EASL Clinical Practice Guidelines on acute-on-chronic liver failure. J. Hepatol..

[4] Lisman T., Porte R.J. (2010). Rebalanced hemostasis in patients with liver disease: Evidence and clinical consequences. Blood.

[5] Zanetto A., Campello E., Senzolo M., Simioni P. (2024). The evolving knowledge on primary hemostasis in patients with cirrhosis: A comprehensive review. Hepatology.

[6] Fisher C., Patel V.C., Stoy S.H., Singanayagam A., Adelmeijer J., Wendon J., Shawcross D.L., Lisman T., Bernal W. (2018). Balanced haemostasis with both hypo- and hyper-coagulable features in critically ill patients with acute-on-chronic-liver failure. J. Crit. Care.

[7] Singh A.D., Mucha S.R., Lindenmeyer C.C. (2022). Cirrhotic coagulopathy: A rebalanced hemostasis. Cleve Clin. J. Med..

[8] Ferdinande K., Raevens S., Decaestecker J., De Vloo C., Seynhaeve L., Hoof L., Verhelst X., Geerts A., Devreese K.M.J., Degroote H. (2025). Unravelling the coagulation paradox in liver cirrhosis: Challenges and insights. Acta Clin. Belg..

[9] Lisman T., Caldwell S.H., Intagliata N.M. (2022). Haemostatic alterations and management of haemostasis in patients with cirrhosis. J. Hepatol..

[10] Lisman T., Arefaine B., Adelmeijer J., Zamalloa A., Corcoran E., Smith J.G., Bernal W., Patel V.C. (2021). Global hemostatic status in patients with acute-on-chronic liver failure and septics without underlying liver disease. J. Thromb. Haemost..

[11] Lisman T. (2023). How to assess hemostasis in patients with severe liver disease. Hematology.

[12] Mitchell O., Feldman D.M., Diakow M., Sigal S.H. (2016). The pathophysiology of thrombocytopenia in chronic liver disease. Hepat. Med..

[13] Scharf R.E. (2021). Thrombocytopenia and Hemostatic Changes in Acute and Chronic Liver Disease: Pathophysiology, Clinical and Laboratory Features, and Management. J. Clin. Med..

[14] Driever E.G., Muntz I., Patel V., Adelmeijer J., Bernal W., Koenderink G.H., Lisman T. (2023). Fibrin clots from patients with acute-on-chronic liver failure are weaker than those from healthy individuals and patients with sepsis without underlying liver disease. J. Thromb. Haemost..

[15] Desborough M.J.R., Kahan B.C., Stanworth S.J., Jairath V. (2017). Fibrinogen as an independent predictor of mortality in decompensated cirrhosis and bleeding. Hepatology.

[16] Lin S., Wang M., Zhu Y., Dong J., Weng Z., Shao L., Chen J., Jiang J. (2015). Hemorrhagic Complications Following Abdominal Paracentesis in Acute on Chronic Liver Failure: A Propensity Score Analysis. Medicine.

[17] Budnick I.M., Davis J.P.E., Sundararaghavan A., Konkol S.B., Lau C.E., Alsobrooks J.P., Stotts M.J., Intagliata N.M., Lisman T., Northup P.G. (2021). Transfusion with Cryoprecipitate for Very Low Fibrinogen Levels Does Not Affect Bleeding or Survival in Critically Ill Cirrhosis Patients. Thromb. Haemost..

[18] Carll T., Wool G.D. (2020). Basic principles of viscoelastic testing. Transfusion.

[19] Mallett S.V., Chowdary P., Burroughs A.K. (2013). Clinical utility of viscoelastic tests of coagulation in patients with liver disease. Liver Int..

[20] Premkumar M., Kulkarni A.V., Kajal K., Divyaveer S. (2022). Principles, Interpretation, and Evidence-Based Role of Viscoelastic Point-of-Care Coagulation Assays in Cirrhosis and Liver Failure. J. Clin. Exp. Hepatol..

[21] Seeßle J., Löhr J., Kirchner M., Michaelis J., Merle U. (2020). Rotational thrombelastometry (ROTEM) improves hemostasis assessment compared to conventional coagulation test in ACLF and Non-ACLF patients. BMC Gastroenterol..

[22] Zhu Z., Yu Y., Ke Y., Deng D., Zheng G., Hua X., Gao G. (2020). Thromboelastography maximum amplitude predicts short-term mortality in patients with hepatitis B virus-related acute-on-chronic liver failure. Exp. Ther. Med..

[23] Premkumar M., Saxena P., Rangegowda D., Baweja S., Mirza R., Jain P., Bhatia P., Kumar G., Bihari C., Kalal C. (2019). Coagulation failure is associated with bleeding events and clinical outcome during systemic inflammatory response and sepsis in acute-on-chronic liver failure: An observational cohort study. Liver Int..

[24] Bihari C., Patil A., Shasthry S.M., Baweja S., Kumar G., Sarin S.K. (2020). Viscoelastic test-based bleeding risk score reliably predicts coagulopathic bleeding in decompensated cirrhosis and ACLF patients. Hepatol. Int..

[25] Lisman T., Bongers T.N., Adelmeijer J., Janssen H.L., de Maat M.P., de Groot P.G., Leebeek F.W. (2006). Elevated levels of von Willebrand Factor in cirrhosis support platelet adhesion despite reduced functional capacity. Hepatology.

[26] Tripodi A., Primignani M., Lemma L., Chantarangkul V., Mannucci P.M. (2013). Evidence that low protein C contributes to the procoagulant imbalance in cirrhosis. J. Hepatol..

[27] Lisman T. (2020). Interpreting Hemostatic Profiles Assessed with Viscoelastic Tests in Patients with Cirrhosis. J. Clin. Gastroenterol..

[28] Campello E., Zanetto A., Bulato C., Maggiolo S., Spiezia L., Russo F.P., Gavasso S., Mazzeo P., Tormene D., Burra P. (2021). Coagulopathy is not predictive of bleeding in patients with acute decompensation of cirrhosis and acute-on-chronic liver failure. Liver Int..

[29] Stravitz R.T., Fontana R.J., Meinzer C., Durkalski-Mauldin V., Hanje A.J., Olson J., Koch D., Hamid B., Schilsky M.L., McGuire B. (2021). Coagulopathy, Bleeding Events, and Outcome According to Rotational Thromboelastometry in Patients With Acute Liver Injury/Failure. Hepatology.

[30] Lisman T. (2017). Decreased Plasma Fibrinolytic Potential As a Risk for Venous and Arterial Thrombosis. Semin. Thromb. Hemost..

[31] Blasi A., Patel V.C., Adelmeijer J., Azarian S., Hernandez Tejero M., Calvo A., Fernández J., Bernal W., Lisman T. (2020). Mixed Fibrinolytic Phenotypes in Decompensated Cirrhosis and Acute-on-Chronic Liver Failure with Hypofibrinolysis in Those With Complications and Poor Survival. Hepatology.

[32] Lebreton A., Sinegre T., Lecompte T., Talon L., Abergel A., Lisman T. (2020). Thrombin Generation and Cirrhosis: State of the Art and Perspectives. Semin. Thromb. Hemost..

[33] Wan J., Roberts L.N., Hendrix W., Konings J., Ow T.W., Rabinowich L., Barbouti O., de Laat B., Arya R., Patel V.C. (2020). Whole blood thrombin generation profiles of patients with cirrhosis explored with a near patient assay. J. Thromb. Haemost..

[34] Zanetto A., Campello E., Bulato C., Willems R., Konings J., Roest M., Gavasso S., Nuozzi G., Toffanin S., Zanaga P. (2024). Whole blood thrombin generation shows a significant hypocoagulable state in patients with decompensated cirrhosis. J. Thromb. Haemost..

[35] Zanetto A., Campello E., Bulato C., Willems R., Konings J., Roest M., Gavasso S., Nuozzi G., Toffanin S., Burra P. (2024). Impaired whole blood thrombin generation is associated with procedure-related bleeding in acutely decompensated cirrhosis. J. Hepatol..

[36] Akyol O., Akyol S., Chen C.H. (2016). Update on ADAMTS13 and VWF in cardiovascular and hematological disorders. Clin. Chim. Acta.

[37] Prasanna K.S., Goel A., Amirtharaj G.J., Ramachandran A., Balasubramanian K.A., Mackie I., Zachariah U., Sajith K.G., Elias E., Eapen C.E. (2016). Plasma von Willebrand factor levels predict in-hospital survival in patients with acute-on-chronic liver failure. Indian. J. Gastroenterol..

[38] van den Boom B.P., Stamouli M., Timon J., Bernal W., Blasi A., Adelmeijer J., Fernandez J., Lisman T., Patel V.C. (2023). Von Willebrand factor is an independent predictor of short-term mortality in acutely ill patients with cirrhosis. Liver Int..

[39] Jachs M., Hartl L., Simbrunner B., Bauer D., Paternostro R., Scheiner B., Schwabl P., Stättermayer A.F., Pinter M., Eigenbauer E. (2022). Decreasing von Willebrand Factor Levels Upon Nonselective Beta Blocker Therapy Indicate a Decreased Risk of Further Decompensation, Acute-on-chronic Liver Failure, and Death. Clin. Gastroenterol. Hepatol..

[40] Enomoto M., Takaya H., Namisaki T., Fujinaga Y., Nishimura N., Sawada Y., Kaji K., Kawaratani H., Moriya K., Akahane T. (2022). Ratio of von Willebrand factor antigen to ADAMTS13 activity is a useful biomarker for acute-on-chronic liver failure development and prognosis in patients with liver cirrhosis. Hepatol. Res..

[41] Takaya H., Namisaki T., Enomoto M., Kubo T., Tsuji Y., Fujinaga Y., Nishimura N., Kaji K., Kawaratani H., Moriya K. (2023). The Ratio of von Willebrand Factor Antigen to ADAMTS13 Activity: Usefulness as a Prognostic Biomarker in Acute-on-Chronic Liver Failure. Biology.

[42] Mahmud N., Hubbard R.A., Kaplan D.E., Taddei T.H., Goldberg D.S. (2020). Risk prediction scores for acute on chronic liver failure development and mortality. Liver Int..

[43] Trebicka J., Fernandez J., Papp M., Caraceni P., Laleman W., Gambino C., Giovo I., Uschner F.E., Jimenez C., Mookerjee R. (2020). The PREDICT study uncovers three clinical courses of acutely decompensated cirrhosis that have distinct pathophysiology. J. Hepatol..

[44] Trebicka J., Aguilar F., Queiroz Farias A., Lozano J.J., Sánchez-Garrido C., Usón-Raposo E., de la Peña-Ramirez C., Sidorova J., Curto-Vilalta A., Sierra-Casas P. (2025). Gene score to quantify systemic inflammation in patients with acutely decompensated cirrhosis. Gut.

[45] Zanetto A., Pelizzaro F., Campello E., Bulato C., Balcar L., Gu W., Gavasso S., Saggiorato G., Zeuzem S., Russo F.P. (2023). Severity of systemic inflammation is the main predictor of ACLF and bleeding in individuals with acutely decompensated cirrhosis. J. Hepatol..

[46] Zanetto A., Pelizzaro F., Mion M.M., Bucci M., Ferrarese A., Simioni P., Basso D., Burra P., Senzolo M. (2023). Toward a more precise prognostic stratification in acute decompensation of cirrhosis: The Padua model 2.0. United Eur. Gastroenterol. J..

[47] Morrison M., Artru F. (2023). Predicting the development of acute-on-chronic liver failure. United Eur. Gastroenterol. J..

[48] Morrison M.A., Artru F., Trovato F.M., Triantafyllou E., McPhail M.J. (2025). Potential therapies for acute-on-chronic liver failure. Liver Int..

[49] Artzner T., Bernal W., Belli L.S., Conti S., Cortesi P.A., Sacleux S.C., Pageaux G.P., Radenne S., Trebicka J., Fernandez J. (2022). Location and allocation: Inequity of access to liver transplantation for patients with severe acute-on-chronic liver failure in Europe. Liver Transpl..

[50] Angeli P., Bernardi M., Villanueva C., Francoz C., Mookerjee R.P., Trebicka J., Krag A., Laleman W., Gines P. (2018). EASL Clinical Practice Guidelines for the management of patients with decompensated cirrhosis. J. Hepatol..

[51] Villa E., Cammà C., Marietta M., Luongo M., Critelli R., Colopi S., Tata C., Zecchini R., Gitto S., Petta S. (2012). Enoxaparin prevents portal vein thrombosis and liver decompensation in patients with advanced cirrhosis. Gastroenterology.

[52] Intagliata N.M., Henry Z.H., Shah N., Lisman T., Caldwell S.H., Northup P.G. (2014). Prophylactic anticoagulation for venous thromboembolism in hospitalized cirrhosis patients is not associated with high rates of gastrointestinal bleeding. Liver Int..

[53] Shatzel J., Dulai P.S., Harbin D., Cheung H., Reid T.N., Kim J., James S.L., Khine H., Batman S., Whyman J. (2015). Safety and efficacy of pharmacological thromboprophylaxis for hospitalized patients with cirrhosis: A single-center retrospective cohort study. J. Thromb. Haemost..

[54] Dane K., Chaturvedi S. (2018). Beyond plasma exchange: Novel therapies for thrombotic thrombocytopenic purpura. Hematol. Am. Soc. Hematol. Educ. Program..

[55] Chen J., Reheman A., Gushiken F.C., Nolasco L., Fu X., Moake J.L., Ni H., López J.A. (2011). N-acetylcysteine reduces the size and activity of von Willebrand factor in human plasma and mice. J. Clin. Invest..

[56] Tersteeg C., Roodt J., Van Rensburg W.J., Dekimpe C., Vandeputte N., Pareyn I., Vandenbulcke A., Plaimauer B., Lamprecht S., Deckmyn H. (2017). N-acetylcysteine in preclinical mouse and baboon models of thrombotic thrombocytopenic purpura. Blood.

[57] Solà E., Pose E., Campion D., Piano S., Roux O., Simon-Talero M., Uschner F., de Wit K., Zaccherini G., Alessandria C. (2021). Endpoints and design of clinical trials in patients with decompensated cirrhosis: Position paper of the LiverHope Consortium. J. Hepatol..

